# Role of ascomycete and basidiomycete fungi in meeting established and emerging sustainability opportunities: a review

**DOI:** 10.1080/21655979.2023.2184785

**Published:** 2023-04-27

**Authors:** Wan Hanna Melini Wan Mohtar, Wan Abd Al Qadr Imad Wan-Mohtar, Afnan Ahmadi Zahuri, Mohamad Faizal Ibrahim, Pau-Loke Show, Zul Ilham, Adi Ainurzaman Jamaludin, Muhamad Fazly Abdul Patah, Siti Rokhiyah Ahmad Usuldin, Neil Rowan

**Affiliations:** a Department of Civil Engineering, Faculty of Engineering and Built Environment, Universiti Kebangsaan Malaysia (UKM), 43600 UKM Bangi, Selangor, Malaysia; b Environmental Management Centre, Institute of Climate Change, Universiti Kebangsaan Malaysia, 43600 UKM Bangi, Selangor, Malaysia; c Functional Omics and Bioprocess Development Laboratory, Institute of Biological Sciences, Faculty of Science, Universiti Malaya, Kuala Lumpur, Malaysia; d Research Institutes and Industry Centres, Bioscience Research Institute, Technological University of the Shannon, MidlandsMidwest, Westmeath, Ireland; e Department of Bioprocess Technology, Faculty of Biotechnology and Biomolecular Sciences, Universiti Putra Malaysia, Serdang, Malaysia; f Department of Chemical and Environmental Engineering, Faculty of Science and Engineering, University of Nottingham Malaysia, Semenyih, Malaysia; g Environmental Science and Management Program, Institute of Biological Sciences, Faculty of Science, Universiti Malaya, Kuala Lumpur, Malaysia; h Department of Biological and Environmental Engineering, College of Agriculture and Life Sciences, Cornell University, Ithaca, NY, USA; i Department of Chemical Engineering, Faculty of Engineering, Universiti Malaya, Kuala Lumpur, Malaysia; j Agro-Biotechnology Institute, Malaysia, National Institutes of Biotechnology Malaysia, Serdang, Selangor, Malaysia

**Keywords:** Fungal biomass, health, food security, water management, innovative designs, wealth and economy, sustainable development goals

## Abstract

Fungal biomass is the future’s feedstock. Non-septate Ascomycetes and septate Basidiomycetes, famously known as mushrooms, are sources of fungal biomass. Fungal biomass, which on averagely comprises about 34% protein and 45% carbohydrate, can be cultivated in bioreactors to produce affordable, safe, nontoxic, and consistent biomass quality. Fungal-based technologies are seen as attractive, safer alternatives, either substituting or complementing the existing standard technology. Water and wastewater treatment, food and feed, green technology, innovative designs in buildings, enzyme technology, potential health benefits, and wealth production are the key sectors that successfully reported high-efficiency performances of fungal applications. This paper reviews the latest technical know-how, methods, and performance of fungal adaptation in those sectors. Excellent performance was reported indicating high potential for fungi utilization, particularly in the sectors, yet to be utilized and improved on the existing fungal-based applications. The expansion of fungal biomass in the industrial-scale application for the sustainability of earth and human well-being is in line with the United Nations’ Sustainable Development Goals.

## Highlights

- Subject-based thematic review of fungal biomass usage and development
- Practical application of fungal biomass aligns with 3 Sustainable Development Goals
- High performance is reported in medical, water management, buildings, and biofuel fields
- Fungal biomass is the lucrative, essential, and future’s way forward

## GRAPHICAL ABSTRACT

**Figure:**
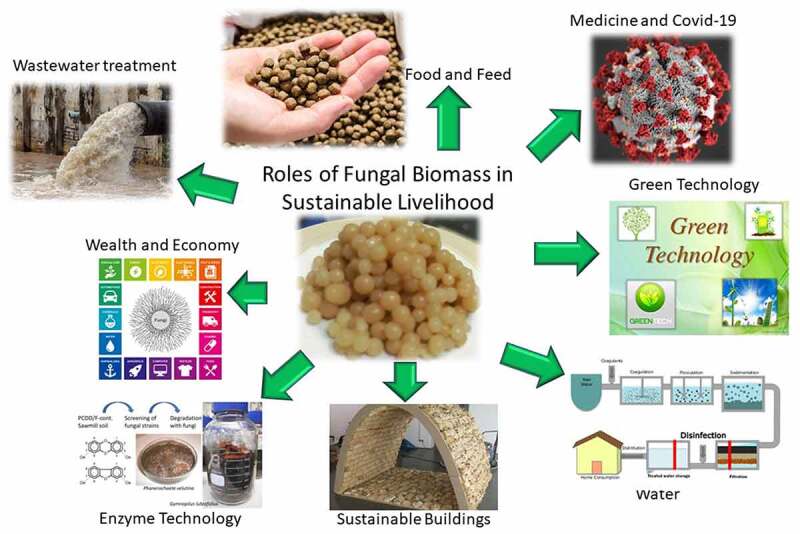

## 1. Introduction

The biodiversity of our world is massive, with an estimation of about 1.5 M species of fungi [1], and about 3.5 to 5.1 M [2], including yeasts, rusts, smuts, mildews, molds and mushrooms. About more than 700,000 species of fungi have been discovered and can be exploited for the benefit of both society and the environment, with more than 3,000,000 species (based on O’Brien’s estimation) waiting to be unearthed, which probably will take about 4000 years [3]. The ubiquitously found fungi, often described as the “fifth kingdom of life on earth remind us of how our planet’s biodiversity is critical in maintaining the health and wellbeing of the world’s inhabitants. Successful applications have been reported worldwide in medicine to combat human disease, plant disease controls, food and beverages processing, biological control, biofertilizers, and industrial production, including fermentation and the paper industry. This article gives primary focus on both ascomycetes and basidiomycetes fungi, giving high potential as bio-alternative with less impact on the ecosystems.

The birth of fungal-based biotechnology started about 100 years ago with the production of citric acid for commercial use in food and beverages and pharmaceutical products [4]. Since then, booming fungal technology has been pivotal in our livelihoods, proving to be a lucrative bio-alternative to petroleum-based products [5]. Hyde et al., (2019) laid out 50 ways to industrially exploit fungi and provide an introduction to the suitability of fungal species to specific industries and technology. The application of fungi as bioresources can be exploited in the circular agricultural economy [6], in line with the aspiration of Sustainable Management Materials (SMM) pursued by the Environmental Protection Agency (EPA). Believing in the crucial roles of fungi now and in the future, this paper compiles the detailed performance of specific fungi strains in distinct industries. We include the latest contributions of fungi in the fields of medicine, production of enzymes, food and feed, and biofuel, with a detailed description of the expanding usage in water and wastewater treatment and sustainable buildings. The possible utilization of fungi now increased to more than 50 ways. We also discuss how fungi shape the world economy, summarizing the innovation of fungi in diverse industries and markets.

With increasing demands on natural-based components over synthetic chemicals, fungi are an attractive alternative biomaterial and lately have received attention. The keyword ‘fungi’ in the title and abstract has brought 60,224 documents (to date) in the academic-based Scopus engine search. Interest in the sustainable biotechnical process has sparked the possible expansion of initially small market segments in the future. Three different levels of science, community and personnel are the keys in relation to human welfare and sustainable fungal biology and biotechnology [7]. Scientific communities have conducted extensive research and development to assess the performance and methods to sustainably exploit fungi, providing critical information to be one step closer to the full-scale fungi utilization in other industrial sectors. Improved understanding of the underlying science also translates into more competent, interdisciplinary, trained personnel in the field. These two top criteria proved to be critical in the European bioeconomy for their long-term sustainable growth and head the fungal-based application at a global scale [8].

This review summarizes the current trend of fungi applications in the selected strategic industrial sectors, improved methods, and associated performance, which we believe are crucial and representative of the fungi’s role in shaping a better future.

## 2. β-Glucans from fungal biomass: implications for health

Taxonomists have described over 148,000 different species of fungi as of 2020. However, it is unclear how diverse the entire fungal kingdom is globally [9]. High throughput sequencing studies estimate there may be 5.1 million fungal species [3]. Fungi have been recognized as both beneficial to human health and wellbeing, along with causing disease [10–12]. Growing interest has been shown in the potential of medicinal mushrooms to improve pulmonary, cardiovascular and anti-cancer health through immune-priming or modulatory properties mostly attributable to constituent beta-glucan molecules [13], which are extracted from fungal biomass [14]. Several plants and foods, including mushrooms, contain complex polysaccharides called β-glucans [12,15]. Structurally, β-glucans are comprised of β-D-glucose monomer units that are held together by glycosidic linkages at different positions (1,3), (1,4) or (1,6). This structure can be either branched or unbranched where the monosaccharide units interconnect at several different points to form a wide variety of different branched and linear structures. Previous research has reported that the variance in glycosidic linkages, molecular weight, branching, degree of polymerization, and solubility can potentially influence function in terms of health benefits [16]. Murphy et al. [17] noted that the therapeutic potential of β-glucans is evidenced by the fact that two glucan isolates were licensed as drugs in Japan as immune-adjuvant therapy for the treatment of cancer. There has been a considerable volume of published research that highlights the potential therapeutic properties of β-glucans including metabolic and gastro-intestinal effects, cholesterol reduction, obesity and diet regulation, cardiovascular and diabetes risk reduction, cancer reduction, bloodstream and wound healing [12,15,18,19]. Due to their potential immune-modulating properties, β-glucans are also being studied as adjuvant treatments for cancer (solid and hematological malignancies), immune-mediated diseases (such as allergic rhinitis and respiratory infections), and to speed up the healing of wounds [15]. Consequently, β-glucans are being tested for clinical efficacy in clinical trials for a plethora of therapeutic applications, including inflammatory conditions, cardiometabolic diseases, obesity, and cancer [15,17]. Indeed, Murphy et al. [17] reported that there are over 200 clinical trials on the therapeutic use of β-glucans that are either completed or in progress. However, these authors noted that as the majority of clinical trial studies administer β-glucans in combination with anticancer drugs or as part of monoclonal antibody treatment, it remains difficult to attribute an effect specifically to β-glucan. Administered doses vary depending on the source of β-glucan and administration route; for example, 1 or 2 mg of β-glucan extract (Lentinan) is administered intravenously to patients with advanced or recurrent stomach, colorectal, and breast cancers [20]. The US Food and Drug Administration approved the use of β-glucan from oat bran for cholesterol-reducing foods with a recommended daily dose of 3 g of β-glucan. Murphy et al. [10] investigated a broad range of commercially marketed products and found them to either contain either no or very little β-glucans based upon established methods.

Mushrooms exhibit a high degree of variability in β-glucan composition that can potentially affect therapeutic functionality. For example, work carried out by senior author demonstrated that two β-glucan extracts from the same Shiitake mushroom can exhibit different effect profiles in an in-vitro lung injury model [21]. Additionally, β-glucan extracts from a commercial source of Shiitake and from an in-house method can significantly affect the ability to clear or reduce clinical isolates of Klebsiella pneumoniae exhibiting multiple antibiotic resistance in an in vivo infection model [13]. Despite their therapeutic potential, as attested by the considerable number of pre-clinical published studies, significant challenges exist to further advance clinical testing and for the reliable and repeatable translation of β-glucans as a therapy [17]. For example, important differences appear to exist in the effects of apparently similar β-glucan preparations that may be attributed to differences in fungal sources and extraction methods, which is also remains poorly understood [13]. From preclinical and clinical trial perspectives, the translational approach to using β-glucans remains heterogeneous in application where greater harmonization of data is required in order to fully elucidate routes of administration, dose, time point, length of treatment and so forth that has led to apparently conflicting findings [15]. This is also further complicated by variations in approaches to β-glucan extraction and purification [14,15]. Such an approach can be used as test reference for comparing and contrasting potential therapeutic effects using β-glucans from other fungal sources. Thus, a potential solution to this challenge would be to isolate β-glucans from fungi have reached an agreed consensus on standard methodologies that encompasses all of the above factors [17]. Recent research has provided in vitro data on the potential of β-glucans to be potentially used as a combination therapy to address complex health challenges such as COVID-19 [15,22] and to combat antimicrobial drug resistance against key bacterial pathogens [13]. However, these approaches have not as yet advanced to clinical trial [18,23].

A focus on medicinal fungi potentially aligns with the search for novel therapeutics and co-therapeutics in the specific field of sepsis [13]. Such solutions require a broad base of source materials that fit multiple criteria, for example, cost, availability, scalability, safety, and potential effectiveness [24]. Developing an improved in-vitro screening process will drive the selection of specific molecules and combinations that progress to inform in-vivo testing, where such a funneling approach will reduce reliance on the use of in vivo infection models [13]. On a related issue, antimicrobial resistance has reached a crisis point [25]. Suitability of new fungal bioactive for established and emerged One Health applications and delivery are diverse, including acute sepsis treatments in combination with antibiotics to reduce resistance issues [13]. In addition, a Quadruple Helix Hub (academic-industry-government-society) framework would accelerate knowledge sharing for solutions with a sustainable focus [26]. Recent research has also highlighted the importance of developing green eco-innovation, including using fungi, in order to unlock complex societal challenges, including disease mitigation [27,28].

## 3. Enzyme technology

Enzyme technology is the application of enzymes in industries and daily life. An enzyme is a biological catalyst produced and carries an important role in the metabolism of all living organisms [29]. The capability of an enzyme to conduct various reactions has led to the exploitation of this biological material to be used in industries and daily life. The utilization of enzymes in the industry has reduced the operational cost by reducing the energy consumption and reaction time while at the same time increasing the yield, process efficiency, and product quality. Besides, utilizing enzymes in industries can reduce our dependency on chemicals, thus, making the process more natural and environmentally friendly [30].

Fungi produce enzymes for their nutrient uptake and metabolism reactions. Fungi secrete extracellular enzymes to break down complex organic materials and predigest the polymeric nutrients into simpler forms to allow them to absorb the nutrients for their growth. Most extracellular enzymes are primary metabolites produced during the log phase, which is essential for their growth [31]. Enzymes produced by fungi are capable of conducting various reactions to degrade and convert substrates to various kinds of products. Therefore, fungal enzymes have been explored and utilized for various industrial applications, especially in the current focus on sustainable global development.

Most fungal enzymes used in the industry are hydrolytic, being recruited to produce more than 700 commercial products [31]. In line with the Sustainable Development Goals, many researchers focus on the cost-effective production of fungal enzymes by utilizing waste materials as substrates. Fungi are capable of degrading various types of substrates, especially organic materials from the agricultural industry, and consuming them for their own growth. However, the efficiency and type of enzymes produced depend on the fungal species, type of substrate, and fermentation operation.

The selection of fungal species for enzyme production depends on the enzymes of interest. For example, amylase can be produced by *Aspergillus niger*, *Rhizopus stolonifer* [32], *Thermomyces lanuginosus* [33], *Paecilomyces variotii* [34], *Rhizopus oryzae* [35], *Aspergillus terreus* [36], *Rhizomucor miehei* [37], *Neurospora intermedia* [38], *Aspergillus awamori* [39], *Aspergillus fumigatus* [40] and other fungal species. The enzyme of á-Amylase produced by *Aspergillus oryzae* is the first microbial amylase used for industrial application [41]. Pullulanase, a group of amylases, can also be produced by fungi, i.e. *Aspergillus sp*. [42], although this enzyme was first discovered in bacteria by Bender and Wallenfels in 1961 [43]. The genus of *Aspergillus* is also capable of producing cellulase [44], glucose oxidase, pectinase [45], mannanase [46], lipase [47], pectinase [48], and other types of enzymes. Another remarkable fungal species employed for industrial enzyme production is *Trichoderma reesei*, which has mostly been used to produce industrial cellulase for over 70 years [49]. Other fungal enzymes produced by different species using various kinds of wastes as substrate are summarized in Table 1.

**Table 1.** Fungal enzymes produced from different types of waste material.

Enzyme	Fungi	Substrate	Production condition				Enzyme activity	References
			Process	pH	Temp (°C)	Duration (day)		
Amylase	*Aspergillus terreus*	1.5% of pomegranate peel waste	SmF	6.0	30	5	339 U/mL	[36]
Pullulanase	*Aspergillus sp*	5 g of wheat bran	SSF	6.0	28.6	5	396 U/g dry substrate	[42]
Pectinanse	*Aspergillus niger*	4 g of citrus waste peel	SSF	5.0	30	5	117.1 μM/mL/min	[50]
Cellulase	*Aspergillus niger* CKB	textile waste	SSF	7.3	28	5	1.56 FPU/g	[51]
Phytase	*Thermoascus aurantiacus* SL16W	5 g of rice bran	Semi SSF	NS	45	9	84.1 U/g substrate	[52]
Lipase	Soil fungal isolate	5 g mustard oil cake	SSF	7.0	NS	NS	7.99 IU/mL/min	[53]
Protease	*Aspergillus niger* WA 2017	Basal medium containing 5 g/L of casein and 5 g/L of peptone	SmF	8.0	30	6	262.9 U.mL	[54]
Lignin peroxidase	*Ganoderma lucidum* IBL-05	5 g of wheat straw	SSF	NS	30	8	2492 U/mL	[55]
Aryl alcohol oxidase	recombinant *Aspergillus nidulans*	Corn steep liquor	SmF	6.5	37	3	1021 U/L	[56]
Lipase	Penicillium chrysogenum	5 g of grease:wheat bran:Czapek-dox media at 1:1:2 (w/w/v)	SSF	7.0	32	8	46 U/mL	[57]

Genetically modified fungi are also being employed to produce high-activity enzymes. Aryl alcohol oxidase (AAO), an extracellular enzyme responsible for lignin degradation, was produced by recombinant *Aspergillus nidulans*. *A. nidulans* was genetically modified by inserting a marker *pyroA4* to prevent the cells from producing pyridoxine. It was genetically modified using DNA-mediated transformation of a plasmid (pEXPYR) inserted into the genome of *A. nidulans*, which resulted in overexpression of AAO [56]. The deletion of small GTPase *rac1* in *Trichoderma reesei* resulted in higher cellulase production. This deletion triggered the hyperbranching phenotype of the fungus, which can be observed in the strong apolar growth during germination and in mature hyphae [58].

In sustaining global development, enzyme production using low-cost and sustainable substrates has been put into focus. Naturally, fungi are capable of digesting and consuming various types of organic materials. Therefore, the utilization of organic waste especially waste from the agricultural, forestry, and food industry, for enzyme production by fungi provide a good impact in achieving sustainable development goals. This approach could reduce the cost while at the same time helping in managing the organic waste produced in these industries. For example, oil palm biomass produced by the palm oil industry, such as oil palm decanter cake and oil palm empty fruit bunch, has been used to produce cellulase by *Trichoderma asperellum* UPM1 and *Aspergillus fumigatus* UPM2 [59,60]. Same fungal species have been used to produce cellulase from sago pith residue produced from the sago industry in Sarawak, Malaysia [61]. As shown in Table 1, notably, various agricultural and industrial wastes such as rice bran, rice straw, wheat bran, wheat straw, mustard oil cake, fruit peel, textile waste, and grease waste have been explored for the production of fungal enzymes, which produce significantly high enzyme activity [62]. This approach could be used to replace processed and pure substrates used in the current enzyme production, potentially reducing the cost and channeling the waste to useful applications.

Two main fermentation operations that can be used to produce fungal enzymes are solid-state fermentation (SSF) and submerged fermentation (SmF). In SSF, a fermentation process is carried out in the absence of free-flowing liquid, whereby the non-soluble material medium acts as a source of nutrients and supportive material to make the medium in the form of a solid [63,64]. The moisture content of 30–85% is usually supplied to support the fungal growth. Technically, SSF mimics the original growing condition of most fungi on organic materials. It was reported that enzymes produced through SSF are higher than SmF. This is because SSF provides higher biomass production, lower protein breakdown, higher enzyme yield, and lower proteolysis as compared to those in SmF [65]. SmF was involved in the inoculation of fungi in a liquid medium [66]. Although the carbon source is insoluble solid biomass, the medium was prepared with a high amount of water that made the solid biomass free-flowing in the liquid medium [67]. The major challenges when using SmF to culture fungi for enzyme production are the reproducibility of the fungi due to mass transfer of oxygen and the proteolysis effect. However, SmF is a more established fermentation operation in the industry with the capability to easily control and monitor pH, temperature, agitation, mixing or aeration, and nutrient supplies [68]. Growing fungi to produce the enzyme in the reactor is usually conducted in a controlled system with a temperature of around 30°C, pH of 6 to nearly neutral, and fermentation duration might take place from 5 days to 9 days (Table 1) depending on the fungal species and the enzyme of interest.

There are huge applications of fungal enzymes in sustaining our livelihood. Enzymes are being used in many industrial processes and daily products. Fungal cellulase is being used in the making of paper, textiles, and detergent for various cleaning purposes; producing juices, bakery, wines; helping in the fermentation process; conversion of cellulosic material to fermentable sugars; animal feed; and many more [69]. For example, fungal cellulase is used to improve the extraction of valuable compounds such as color or beta-carotene from the vegetable mash, improve juice yields, and cloud stability of the extracted juice. Fungal pectinase is used to improve juice filtration in cross-flow membrane filtration systems by completely removing pectin and arabinan, thus improving cross-flow membrane filtration. High UF-Flux and long filter runs and extending membrane shelf life. Cellulase and xylanase produced from ground palm cake are able to improve the oil separation from the decanter effluent [70]. Fungal enzymes such as lipase produced from grease waste have the potential to bioremediate used cooking oil [71], á-Amylase produced by *Aspergillus oryzae* is used in the brewery industry to cleave maltose to sugar, which accelerates the fermentation process and thus reduces fermentation duration [72]. á-Amylase is also applied in the bakery industry to improve dough, make the crust color and texture better, and shorten the fermentation time [73].

Overall, fungal enzymes are very important in providing a beneficial impact on our livelihood. The utilization of fungal enzymes also helps in achieving the global sustainable development goals, especially when the production of fungal enzymes employs waste materials as the substrate. Because enzymes are frequently present at very low concentrations with many impurities, similar properties to those of the product and contaminants, and poorly characterized properties with respect to physiochemical characters and thermodynamic properties, the enzyme purification process poses a significant technical challenge.

## 4. Food and feed

Fungal biomass, primarily from Ascomycetes and Basidiomycetes, has been incorporated into the dietary needs for both humans and animals. They are a valuable group of organisms in terms of nutrition, economics, and biotechnology [74,75]. They are nutritionally dense, with low calories, high protein, and high fiber content. Aside from being a valuable source of food, fungal biomass is used by humans in a variety of ways. Mushroom biomass, which is classified as 200 superfood species [76,77], can be employed as anti-cancer, antioxidant, and immunomodulating agents in the pharmaceutical business [78]. They are advantageous for both people and forests because they serve as natural decomposers and nutrient recyclers [79]. Almost all terrestrial multicellular organisms have mutualistic relationships with fungi, which break down decaying plant material. In forests and other ecosystems, when organic matter is not present, decomposer fungus flourishes. Meanwhile, non-mushroom biomass is used for heavy metal biosorption [80,81], dye decolorization [82,83], biodiesel production [84,85], and pigment synthesis [86].

Because of its high nutritional value, fungal biomass has been included in human diets. Proteins, minerals, B vitamins, vitamin D, vitamin K, and rarely vitamins C and A are all found in cultivated fungal biomass, which is typically low in fat and high in protein [87]. In human societies that do not consume animal proteins, fungal biomass is utilized to supplement plant grains and minimize protein deficits (either due to scarcity or religious beliefs) [88]. Fungal protein provides all nine essential amino acids (EAAs), whereas most other protein sources only contain one or two EAAs. In fact, fungal biomass has a high concentration of branched-chain amino acids (BCAAs), which are normally found solely in animal-based protein sources [89]. The protein content of fungal biomass is higher than that of grains, but it is comparable to that of other non-animal protein sources [90]. Non-digestible carbohydrates found in fungal biomass include -glucans, raffinose, oligosaccharides, chitin, and resistant starch, among others [91].

Table 2 shows how fungal biomass has been introduced into a variety of foods, including patty burgers, steamed buns, cookies, sponge cake, biscuits, tikki, pasta, ketchup, sausage, noodles, and chips. Food companies added mushroom biomass to these foods to increase pharmacological qualities such as cholesterol regulation, tumor-fighting response, blood pressure and blood sugar regulation, and immunity [97]. Calocybe indica-enriched cookies [98] and Shiitake noodles [99] have both shown a drop in the glycemic index, proving these theories. Non-mushroom biomass, on the other hand, has not been individually incorporated into the food source but rather has been formulated with *Saccharomyces cerevisiae*, such as Marmite, Vegemite, and Quorn (*Fusarium venenatum*). These products are typically generated in a continuous fermentation culture system in a large-scale bioreactor [74,95]. Table 2 also reveals that mushroom-derived fungal biomass is more likely to be used in food production than non-mushroom biomass, owing to its safety, high protein content, nontoxic, ease of mass cultivation, and improved flavor [100].

**Table 2.** Common incorporation of fungal biomass (mushrooms and non-mushrooms) into the food source.

Fungal species	Food products	Reference
Mushrooms		
*Pleurotus sapidus*	Cookies, steamed buns, Patty burger	[87,92–94]
*Pleurotus eryngii*	Sponge cake, Chicken burger	
*Pleurotus tuber-regium*	Cookies	
*Pleurotus sajor-caju*	Biscuits, Cookies, Chicken patties	
*Pleurotus ostreatus*	Biscuits, Jam, Soup, Additive powder, Cheese spreads, Instant noodles, Soup premix, beef patties	
*Boletus edulis*	Beef burger	
*Calocybe indica*	Cookies	
*Tremella fuciformis*	Patty, Patties	
*Agaricus bisporus*	Ketchup, Tikki, Pasta, Sponge cake, Meat emulsion, Patties, Fish paste	
*Flammulina velutipes*	Sausage, Tuna meat, Goat meat nuggets	
*Lentinula edodes*	Chips, Noodles, Patties	
Non-mushrooms		[95]
*Saccharomyces cerevisiae*	Marmite, Vegemite	
*Fusarium venenatum*	Quorn	[95,96]

Fungal biomass has been utilized as feed for ruminants, fish, and poultry. It plays an important function in feed production, especially in aquaculture activities such as improving gut microbiota, immunology, growth, antioxidative responses, and lipid profiles [87,101]. For example, mesophilic fungal biomass has recently increased pond soil diversity for freshwater prawn productivity [102]. Table 3 shows how fungal biomass is used in the feed business, mostly through mushrooms such as *Agaricus* sp. (promoting the health of broiler chickens), *Ganoderma lucidum* (increasing the body weight of Red Tilapia), *Pleurotus* sp. (increasing the health of pigs), *Armillariella tabescens* (pigs’ health), *Lentinula edodes* (fish growth), and *Tremella fuciformis* (improved body weight of broiler chickens). Some of the non-mushroom biomass has been classified as Generally Regarded As Safe (GRAS) microorganisms that have been widely used in food creation, including *Rhizopus arrhizus*, *Neurospora intermedia*, and *Aspergillus oryzae* [116]. Karimi et al. [116] used *Rhizopus oryzae* biomass as fish feed to confirm his idea, which enhances gut microbiota growth, growth performance, immunity, and antioxidative response [87].

**Table 3.** Fungal biomass (mushrooms and non-mushrooms) incorporation into feed source.

Fungal species	Animal pellets	Reference
Mushrooms		
*Agaricus blazei*	Broiler chicken	[103]
*Agaricus bisporus*	Broiler chicken	[104]
*Ganoderma lucidum*	Red tilapia, Holstein cow, Finisher pigs, Broiler chicken	[90,105–107]
*Pleurotus ostreatus*	Berkshire pigs, piglets, broiler chickens	[107–111]
*Pleurotus sajor-caju*	Nile tilapia	[111]
*Armillariella tabescens*	Early weaned pigs	[112]
*Lentinula edodes*	Rainbow trout fish, Sturgeon fish, Broiler chicken	[107,113–115]
*Tremella fuciformis*	Broiler chicken	[115]
Non-mushrooms		
*Aspergillus oryzae*	Pig, Fish	[95,116]
*Rhizopus arrhizus*	Pig	[95,116]
*Neurospora intermedia*	Fish	[116]
*Rhizopus oryzae*	Fish	[116]

With low levels of fat, salt, cholesterol, and calories, fungal biomass is becoming recognized as a rich source of bioactive compounds and essential nutrients, such as fibers, proteins, minerals, vitamins, and nutraceuticals, making it suitable for intake by both humans and animals [75]. Additionally, the synthesis of edible fungal biomass can be more environmentally friendly [117,118] when compared to other protein products of both animal and plant origin, leading to fewer adverse effects on the ecosystem. Additionally, this edible fungal biomass’ organoleptic properties [119], such as flavor and texture, make them suitable meat alternatives [120,121]. Although they are thought of as sources of useful functional components, their quick application is advantageous for enhancing a variety of ready-to-cook and ready-to-eat foods as well as animal feed. Furthermore, unknown fungal biomass may have a nutritional, environmental, and sensory influence that differs from that of previously studied species. As a result, there is a pressing need to investigate new or understudied fungus species.

## 5. Water treatment

Domestic water is used for various purposes and is consumed regularly. Surface water and groundwater are two types of water that can be used for home water supply. For home water, all water supplies must meet safety and hygiene regulations. Clean and treated water has long been a concern around the globe, as increasing populations, demands for technology, and industrial operations are seen as factors affecting water security. To address this issue, various innovations and appliances have been installed in most homes to obtain a glass of the purest and safest drinking water possible. The World Health Organization (WHO) has stated that safe drinking water should be free of pathogens as well as dangerous and non-damaging substances that can alter the water’s color, taste, pH, and odor [122].

Before drinking or utilizing safe treated water, it must be pathogen-free or have a low degree of pathogen contamination. When bacteria in the water reach a particular level, they may create an outbreak if taken into the body. According to Table 4, fungi such as *Trametes* sp., *Pleurotus* sp., *Lentinus* sp., and *Agaricus* sp. are used in water treatment investigations. This species is frequently employed since most are white-rot fungi with antibacterial properties. Brown-rot fungi such as *Gloeophyllum trabeum* and *Serpula lacrymans* [129], which contain nearly the same active chemical as white-rot fungus, can prevent the growth of dangerous microorganisms in the water.

**Table 4.** The use of fungi as microorganism removal in water treatment.

No	Fungi Species	Microbes Species/Heavy Metals	Source of Water	Temperature (°C)	Efficacy of Treatment (Microbes Removal/Clear Zone Diameter/Heavy Metal Removal)	Reference
**1**	*Trametes versicolor*	*E. coli*	River water	23–29	24% without mycelium, 28% with mycelium	[123]
**2**	*Pleurotus ostreatus*	*E. coli*	River water	25	99.25% (lab) 99.75% (river water)	[124]
**3**	*Pleurotus tuberregium*	-	Boreholes, Stream, River, Well and Pond drinking water	26	0.0–4.1x10^3 (borehole) 0.00 (stream) 0.4x10^3 (pond) 0.0 (river) 5.80x10^3 (well water)	[125]
**4**	*Lentinus squarrosulus*	*E. coli*	River water	-	100%	[126]
**5**	*Stropharia rugosoannulata*	*E. coli, Raoultella plantiloca, Enterobacter sp.*	Synthetic stormwater	20		[127]
**6**	*Pleurotus tuberregium*	Chromium, Lead, Copper, Nickel, Cadmium, Iron, Zinc, Manganese, Aluminium, Cobalt, Silver, Arsenic	Drinking water from boreholes, streams, rivers, wells, and pond	-	Chromium − 100% (boreholes, stream, pond, river, wetlands), Lead − 100% (boreholes, stream, pond, river, wetlands), Copper − 99% (borehole & wetlands) 100%% (stream, pond, river)	[125]
**7**	*Pleurotus ostreatus, Pleurotus sajor caju, Agaricus campestris, Agaricus bisporus, Grifola frondosa*	*Pseudomonas aeruginosa, Staphylococcus aureus, Shigella sp., E. coli*	-	28	***Pseudomonas aeruginosa*** − 14mm (*Pleurotus ostreatus*), 15mm (*Pleurotus sajor caju*), 25mm (*Agaricus campestris*), 16mm (*Agaricus bisporus*), 20mm (*Grifola frondosa*) ***Staphylococcus aureus*** − 12mm (*Pleurotus ostreatus*), 18mm (*Pleurotus sajor caju*), 30mm (*Agaricus campestris*), 17mm (*Agaricus bisporus*), 23mm (*Grifola frondosa*) ***Shigella sp.*** − 12mm (*Pleurotus ostreatus*), 16mm (*Pleurotus sajor caju*), 18mm (*Agaricus campestris*), 16mm (*Agaricus bisporus*), 21mm (*Grifola frondosa*) ***E. coli*** − 15mm (*Pleurotus ostreatus*), 19mm (*Pleurotus sajor caju*), 21mm (*Agaricus campestris*), 16mm (*Agaricus bisporus*), 21mm (*Grifola frondosa*)	[128]
**8**	*Gloeophyllum trabeum, Serpula lacrymans*	*E. coli*	Synthetic *E. coli* water	37	8.33mg mL (*Gloeophyllum trabeum*), 4.55mg mL (*Serpula lacrymans*)	[129]
**9**	*Pleurotus enryngii*	Fluoride	Fluoride water	2–7	Fluoride − 81.2% (pH 2), 92% (5mgL flouride), 97.03% (0.1g fungi), 97.5%(200-300mins)	
**10**	*Pleurotus enryngii*	Cadmium (II)	Synthetic cadmium water	3–8	Cadmium (II) − 70% (pH5), 95% (0.2g fungi), 98.1% (20mgL Cd)	[130]
**11**	*Pleurotus ostreatus*	Chromium	Synthetic chromium water	5.6	Chromium; 100% in 10mgL, 24.9% in 150mgL (active), 100% in 10mgL, 39.88% in 150mgL (inactive)	[131]

The most common bacteria found in water bodies is *Escherichia coli*, which must not be present in the treated water. It is the most commonly treated bacteria utilizing fungal species, and it is also used as a pollutant and pathogen concentration indicator. According to Table 4, river water has the highest prevalence of *E. coli* [123,124,126]. Other than *E. coli*, *Pseudomonas* sp., *Staphylococcus* sp., and *Enterobacter* sp. have been found in other synthetic water bodies [125,127] because the characteristics of environmental liquids are similar to those of human intestinal fluid [132]. In addition, temperature is the most crucial characteristic in water treatment because most fungi function best at 23–37°C, as described in Table 4.

On an antimicrobial test, the fungal species kills bacteria at an average zone of inhibition of 12–21 mm, which is comparable to the standard manufacturers’ antibiotic disc (21 mm) [133]. *Lentinus squarrosulus* has the maximum microbial removal from the river water sample, with 100% removal, followed by *Pleurotus ostreatus* (99%) and *Trametes Versicolor* (24–28%) [123,124,126]. The laccase concentration, which impacts the potential and ligninolytic enzymatic activity, is the difference in performance between these species [134]. It is stated that 0.25% (w/w) of the polysaccharide from the macrofungi can be the minimum inhibitory concentration (MIC) for phatogenic microbes such as *E.coli* in water [135]. Brown-rot fungi have less enzymatic activity than white-rot fungi; the creation of unpleasant compounds may be involved in the brown-rot fungi’s bactericidal and bacteriostatic activities [136].

A cocktail of contaminants in the water is considered chemical contamination that originated from different sources, such as natural sources, industrial waste activities, and agricultural residues. Because of their treatment efficacy at normal water temperatures (23–37°C), white-rot fungi are the most cost-effective technique to treat chemically contaminated water, according to Table 4. Hence, it is proven that this species can purify raw water for potable uses. *Pleurotus* mushroom is frequently used in water treatment studies because it is easy to cultivate and exhibits significant laccase activity during the mycelium growth phase, resulting in a shorter treatment time [137]. In addition, *Pleurotus sp*. can reduce the concentration of a broad spectrum of heavy metals abundantly present in the water, with *Pleurotus tuberregium* capable of degrading various types of heavy metals (chromium, lead, copper, nickel, and iron) [125]. These fungi are high in proteins, amino acids, polysaccharides, unsaturated fatty acids, minerals, and the hyperbranched β-glucans, which have been discovered to be novel biopolymers that could be used as water-cleaning functional nanomaterials [138]. *Trametes versicolor* is a suitable herbicide and pesticide decomposing diuron [139] because it can synthesize lignin and manganese peroxidases in significant amounts [140].

Fungi can treat and purify water whether it is contaminated or not, as fungi are resistant to the harsh conditions of the environment. According to Table 4, water contaminated with heavy metals has a pH in the range of 2 to 8 [130,131,141,142]. As fungi are pH-versatile, it can grow under a wide range of pH, but most fungi thrive better in a mildly acidic environment, especially wood decaying fungi such as Basidiomycetes. In addition to that, a pH range between 4.5 and 8.3 is optimal for a high yield of polysaccharide production [143]. In contrast, *Agaricus bisporus* can withstand a slightly higher pH of 12 for growth and treatment [144]. In treating water, *Pleurotus ostreatus* and *Pleurotus tuberregium* have the significant potential to treat chromium in water, possibly up to 100% removal [125,131] as it has carboxylic, amino, thiol, phosphate, and hydroxide groups that aid in chemisorption and ion-exchange in metal biosorption on the cell wall. Inactive *Pleurotus sp*. somehow has better adsorption on heavy metals and chemicals in water than using the living biomass of the fungi because dormant fungi biomass has a higher binding site, functional groups, and percentage of heavy metal removal. Moreover, the removal rate is fast compared to the living biomass, requiring low energy demand and cost [145]. These species have varying levels of affinity for different types of heavy metals [146]. In a study on *Agaricus bisporus* (approximately 25.9% to 89% heavy metal removal) [144,147], the phenol and antioxidant concentration in this species is lower than in other species [148]. *Trametes versicolor* is suitable for use as an adsorption agent in drinking water. In hypersaline conditions, it may enhance the absorption rate of fungi by 70% [139]. Manganese peroxidase production is higher and strongly enhanced in hypersaline or extreme conditions.

## 6. Wastewater treatment

In this work, we separately compile and discuss the fungal-based treatments for water and wastewater, with notably more studies having been conducted on wastewater. Treating wastewater traditionally used bacteria-based processes such as activated sludge to reduce the most dominant pollutants in domestic wastewater, ammonia, and organic content. Recent technologies of Moving Bed Biofilm Reactor (MBBR), also utilizing the ability of bacteria, is able to produce a good performance of nutrient removal in domestic wastewater [149]. Although the bacteria process dominated the wastewater treatment, fungal treatment has been explored and produced favorable outcomes, if not better than the bacteria-based methods. Utilizing the ability of fungi to produce high amounts of nonspecific oxidative enzymes, fungal treatment has high performance in degrading pollutants in pesticides, pharmaceutical compounds, industrial chemicals, contaminated water, and textile waste [150]. The versatility and nonspecific enzymatic cocktail of fungi allow for the degradation of wide-range contaminants and have advantageous features over bacterial-based treatment. Several studies have investigated the feasibility of basidiomycetes as alternatives in wastewater treatment [150,151]. Due to their versatile enzymatic machinery, fungal species of *Phanerochaete chrysosporium, Pleurotus ostreatus*, and *Trametes versicolor*, to name a few, are now seen as alternatives to the removal of environmental contaminants [152–154].

Fungal-based treatment has advantages over bacterial processes, notably the ability to degrade pollutants with highly complex structures. The degradation of pollutants is bacteria-specific and becomes less efficient when treating combinations of pollutants. Domestic wastewater contains a cocktail of nutrients (nitrogen and phosphorus), oxygen demanding wastes, organic matter, and micropollutants from antibiotics, hormones, drugs, and caffeine [155]. The versatility and nonspecific enzymatic machinery in fungi allow for the degradation of several contaminants simultaneously [156].

The research of fungi in treating pollutants in wastewater can be said in parallel with the progression discussion of contamination in water resources. Based on the Scopus search, the degradation of pollutants by fungi was first reported to decolorize wastewater using *P.chrysosporium* in the year 1980, achieving 60% color reduction [157]. The use of fungal-based treatment is then expanded to the removal of metal ions [158–160], whereby fungals work well as biosorbents with higher sorption capacities than commonly used activated charcoal and ion-exchange resin. By the early 1990s, the research on decolorization by fungi started to kick off for acid dyes [161], distillery effluent [162] and synthetic dyes [163,164]. Fungal-based treatment is also proven as an attractive alternative for treating polycyclic aromatic hydrocarbons [165] and the contaminants of dye organochlorine compounds [166]. The research on fungi’s capability in wastewater treatment processes receives mass attention entering the 21st century, polishing up more on the optimized conditions for improved pollutant removal.

The past decades have shown increasing attention focused on the fungal-based treatment of contaminants of Emerging Concern (ECs), including pharmaceutical compounds and endocrine-disrupting chemicals (EDCs). ECs, particularly the domestically consumed therapeutic groups, are mostly discharged through sewage treatment plants. Attention has been given to the removal of ECs, pharmaceuticals (and their metabolites) as the conventional wastewater treatment process was not designed to efficiently treat them, resulting in a high probability of discharge (along with the effluent) into the river. They also survive the physical treatment processes at the water treatment plant. Although the consumption of pharmaceutical products varies from one city or country, the recent trend in Malaysia showed an increasing trend of sex hormones and modulators of the genital system (up to 60%) in the year 2016 [167], indicating an increasing prospect of ECs in our waterways. The focus was given as the ECs are reported to be available in our river and treated water, albeit at low concentration, limited established standard guidelines and uncertainties on the impacts of ECs on the human health, river ecosystem, and socioeconomics accentuate the efforts on their removal. Interestingly, nearly complete removal of specific analgesics and anti-inflammatories [168], antibiotics [152,169], psychiatric drugs [170], EDCs [171,172], pesticides [171,173] were reported. Having said that, not all ECs were able to be removed or have a low percentage of reduction [150].

Numerous technologies are available for treating ECs, such as adsorption using activated carbon [174], membrane filtration [175,176], advanced oxidation processes, and biological-based processes [177]. Despite the high reduction capacity in chemical-based treatment, the usage of toxic chemicals and the generation of hazardous by-products pose significant drawbacks in creating an environmentally friendly treatment process. The greener biological processes, such as the activated sludge system, has proven effective in the removal of ECs in water. However, the efficiency of removal depends on the oxygen content, the degradability of ECs and bacteria-specific [178]. Manipulating the advantages of fungi, particularly the extraordinary extracellular enzymatic system, ability to withstand the harsh environment and no hazardous by-products, fungal-based treatment has high potential in treating a wider range of ECs.

It has been demonstrated that using fungal species to remove ECs from wastewater by biotransformation, biodegradation [179], and biosorption [180], as opposed to conventional wastewater treatment technologies, is a good alternative. Fungal pellets are self-immobilizing aggregates that settle easily. Their usage in bioreactors holds promise since it avoids the practical and technological challenges that dispersed mycelium frequently presents, as well as the high operating costs of conventional wastewater procedures. The primary factors encouraging enterprises to employ biological systems for wastewater treatment include their low-cost, high-energy efficiency, and ability to produce valuable by-products that can be used as fertilizers or in the generation of electricity. The capabilities of ascomycetes and basidiomycetes bioremediation have been proven effective on sterile glucose-based spike media (i.e. synthetic) wastewater. However, the feasibility of real wastewater is yet to be fully understood, whereby to advance the technology to an industrial scale, the survival of the fungal community within the complexity and dynamic conditions of real wastewater must be investigated. The stiff competition for getting the substrate with the autochthonous microorganisms in the wastewater leads to loss of fungal biomass and destabilization of fungal enzymes [181]. However, a positive synergistic effect between fungal and bacteria is possible [169] and thus, poses a high potential in utilizing fungal-based domestic wastewater treatment.

On this note, not exactly implying no competition with the autochthonous microorganisms, fungi provide assistance as the carrier for bacteria and algae, enhancing the capacity of pollutant removal. Fungal pellets immobilized denitrifying bacteria of *Pseudomonas stutzeri sp*. GF3 has 100% denitrification efficiency, removing nitrate from wastewater [182]. The bacteria were successfully fixed and distributed on the surface and inside of the fungal pellets, promoting better nitrate removal. The pellet-assisted micro-algae managed to achieve an efficiency of more than 95% (on average), with the retention time spanning between 3 to 72 hours, much shorter than the commonly adopted time of a week. The time to achieve good performance is based on the fungal-algal strain. The commonly used family fungal strains as co-culture with algae are *Aspergillus*, with other strains were also exhibiting good performance in pollutant removal, including *Penicillium, Pleurotus, Trichoderma, Cunninghamella* and *Isaria* [183].

The success of fungal-algal coupling is due to electrostatic neutralization, surface protein interaction, and EPS adhesion, the (micro)algae can co-exist with the fungi by sticking to the fungal hyphae. The bioflocculation of fungi-assisted microalgae has received attention as it has low cost and high performance [184]. The fungi act as carriers, promoting a better environment for harvesting microalgae, increasing the total biomass and giving a higher performance for pollutant removal by microalgae. Having said that, the fungi itself has good capability to degrade pollutants, as exhaustively shown, and should be exploited to work together with bacteria or algae, increasing the performance of pollutant reduction.

Recent studies have investigated the integration or hybrid treatment process to increase the efficiency of pharmaceutically active compounds, coupling fungal-based treatment with the Advanced Oxidation Process (AOPs) [185] or microalgae [183,184]. The coupling approach is able to achieve up to 95% removal of pharmaceutically active compounds using either AOPs/*T.versicolor* or *T.versicolor*/AOPs treatment processes [185]. The coupling *T.versicolor*/AOP also has a high potential to remove the parent pollutant compound (metoprolol), producing fewer 3rd generation intermediates and more 1st and 2nd generation (metoprolol acid) transformation products [186], promoting a more efficient removal.

Initially, the roles of ascomycetes and basidiomycetes were as physical biosorbents. Recent technology has seen that *Phanerochaete chrysosporium* is able to produce soluble microbial product (SMP), acts as a bioflocculant and has better performance in coagulating kaolin than the commercialized polyacrylamide [187]. Due to the bridging mechanism, the high molecular weight polysaccharides in the SMP provide flocculation activity. They comprise hydroxyl and carboxyl groups, the functional groups that effectively cause flocculation and exhibit self-orientation behavior in water.

Interestingly, throughout the literature, the use of fungi in treating wastewater has been exhaustively studied not for domestic wastewater but mostly on the discolouration of textile wastewater and the removal of heavy metals and pharmaceutical compounds. With abundant nutrient content in domestic wastewater, it is envisaged that the fungal treatment would accelerate the pollutant removal, particularly with the presence of domestic-consumed ECs. The performance of the basidiomycete *Ganoderma lucidum* was assessed in the removal of ammonia and organic content in synthetic urban wastewater (expressed in terms of Chemical Oxygen Demand) and was able to achieve about 90% removal after 48 hours [188]. The coupled algae-fungal technology has the best nutrient removal in synthetic domestic wastewater [189], proving the benefit of a synergistic effect of both cultures.

Despite the wide variety of fungi species and strains, the mostly used fungi widely reported to produce a high performance of pollutant removal in wastewater is *T.versicolor* [190], where most of the studies were published in the last decades. Notably, the popular *T.versicolor* strains are from the American Type Culture Collection (*T.versicolor* ATCC#42530) or *T.versicolor* 167/93. Although the efficiency of *T.versicolor* may achieve 100% of removal (or complete degradation), the good performance was not essentially the case for all studied pollutants and depended on the dynamic environmental conditions. Another basidiomycetes that showed good performance are the *Bjerkandera sp. R1*, *Bjerkandera adusta* and *Phanerochaete chrysosporium* [191]. A recent discovery of the halotolerant, high-salt resistant fungal strain *Aureobasidium sp*. MSP8 from the saline-activated sludge in wastewater treatment was able to remove phosphorus from the actual brewery and chemical wastewater up to 54%, a harsh high salinity environment (5%) [192]. The strain also has a wide range of adaptability, particularly pH (between 3–7) and temperature (20–30°C), providing alternatives for phosphorus removal in a saline treatment plant.

Despite the high performance of removal, the biggest drawback of fungal-based treatment is the long retention time needed, on average, about 1–3 days [150] or can go up to 7 days to 14 days [190]. With the current (traditional bacteria-based) treatment process normally taking about 4–6 hours, incorporating fungal pellets into the existing setup poses a challenge. Table 5 provides an overview of retention time using fungal treatment, listed based on the studies published in 2020 and 2021, emphasizing the recent trend of fungal treatment. Although a couple of studies utilized long retention times of up to 8 days, achieving high efficiency on shorter days is feasible. Furthermore, fungi as bioflocculants and biosorbents proved to degrade pollutants within 1 hour. Another major setback in ensuring an efficient fungal treatment is that it requires an acidic environment, preferably at pH 4.5 [188], which is within the optimal pH range for fungal growth. This slight disadvantage could be overcome by methodically reducing the pH with the acid solution, albeit it would increase the treatment cost.

**Table 5.** Main summary of recent studies using the fungal-based treatment for various types of wastewater.

No	Author (year)	Treatment type	Type of wastewater	Targeted compound	Fungal strain	Retention time (days)	Efficiency (max %)
1	Mir-Tutusaus et al. (2021) [185]	Coupling UV/H202 + fungal	real hospital	pharmaceutical (22)	*T.versicolor* ATCC#42530	7	94%
2	Zheng et al. (2021) [182]	fungal pellets	wastewater	nitrate	*Phoma sp* ZJ6	4 to 8	96%
3	Zeng et al. (2021) [192]	fungi cell	saline industrial	phosphorus	*Aureobasidium* sp. MSP8 (isolation from AS)	7	77.20%
			brewery and chemical			7	53.50%
4	Jaén-Gil et al. (2021) [186]	UV/H_2_0_2_ + fungi	hospital	metoprolol acid	*T.versicolor* ATCC#42530		36.40%
5	Li et al. (2021) [187]	soluble microbial products as bioflocculant	municipal	COD	*Phanerochaete chrysosporium*	5 mins and 20 seconds (as bioflocculant)	91.80%
6	Tormo-Budowski et al. (2021) [190]	fungal pellets	hospital	pharmaceutical (16)	*T.versicolor* ATCC#42530	14	95.7% synthetic 85% real
7	Nouri et al. (2021) [193]	fungal biomass	textile	azo dye	*Sarocladium sp*. (dried biomass)	1 hour (as biosorbent)	97.40%
8	Saravanan et al. (2021) [194]	fungal biomass	synthetic	Cu(II) and reactive green 6 dye	*Aspergillus niger* and *Aspergillus flavus*	60 mins	95%
9	Dalecka et al. (2020) [195]	fungal biomass	municipal	pharmaceutical (4)	*Aspergillus luchuensis* (isolation from AS)	3	>99.9%
					*Trametes versicolor* DSM 6401	3	>99.9%
10	Negi et al. (2020) [196]	fungal pellet	synthetic ww (selenite rich)	selenite	*Aspergillus niger* KP	3	94%

## 7. Sustainable buildings

Through the extraction, processing, manufacture, and transportation of construction materials, the building sector contributes to global carbon emissions. These call for a more sustainable strategy for carbon reduction. Composites derived from mycelium have been found as a safe and ecologically friendly alternative to conventional building materials [197]. The mycelium serves as a natural binder and it is made up of individual hyphae that develop from spores of the mycelium fungal strain by consuming carbon and nitrogen-containing food [198]. Mycelium-based composites are biodegradable and low-energy building materials that help protect the environment and reduce waste emissions, particularly through the upcycling of agricultural by-products and wastes [199]. The use of mycelium to construct a living house is a movement that is environmentally friendly, promotes biodiversity and natural equilibrium, and permits sustained and healthy human growth [200]. As a benchmark for sustainability, the Life Cycle Assessment (LCA) of mycelium bio-composites revealed that they emit substantially less CO_2_ than conventional insulators [201]. This is because the manufacturing process utilizes renewable feedstocks that are biodegradable at the conclusion of the product’s life cycle.

Elsacker et al. [202] showed that mycelial materials are circular by the upcycling of lignocellulosic by-products. With all the major elements affecting manufacturing, it exposes the possibility of new, previously unimagined uses. In general, the procedures used to create mycelium-based composites begin with the homogenization of a chosen substrate to enhance the growth surface area, followed by sterilizing to eliminate microbial competition [203]. Then comes fungal colonization, which is an inoculation procedure that involves the use of spores, hyphal tissue, and fruiting body tissue in a controlled environment for specified periods prior to dehydration and characterization [197]. Cerimi et al. [204] conducted a patent study from 2009 to 2018 and discovered 47 patents and patent applications covering 27 distinct fungus species as potential sources of novel bio-based products. These patent discoveries demonstrate that fungus-based materials have the potential to be a good substitute for petroleum-based products.

As a rapidly renewable resource that can grow in any form, it is also financially feasible, rational, and beneficial [200]. Controlled processing procedures, according to Manan et al. [197], enable the creation of mycelium-derived materials with the appropriate function and structure for detailed purposes. This is corroborated by Soh et al. [205], who discovered that a 60:40 or 70:30 mixture of chitosan and mycelium-enriched bamboo, as well as a 3 wt percent chitosan solution made at pH 6, generated workable, extrudable, and buildable mycelium-bound materials capable of sophisticated shape and design. According to Attias et al. [206], material composition and manufacturing circumstances are two elements that should be addressed throughout the architectural design process in relation to the final product’s intended purpose. Surprisingly, the coupling of microbial systems with fibrous substrates results in the formation of a novel class of bioactive materials capable of self-healing damage and bridging gaps while alive [207].

Natural reinforcement particles increased the density of mycelium bio-composites marginally above typical mycelium bio-composites without compromising mycelium development [208]. A mixture of sugar cane and cassava root waste demonstrated favorable performance, with a density of 440 kg/m^3^ and a compressive strength of 0.61 MPa at 5% strain and an average elastic modulus of 22.70 MPa, whereas woodchips and sawdust demonstrated an average compressive strength of 0.17 MPa at 5% strain and an elastic modulus of 3.97 MPa [198]. A combination of millet grain, wheat bran, wood pulp, natural fiber, and calcium sulfate mixed according to a specific protocol resulted in an innovative fungal mycelium-based biofoam with thermal conductivity of 0.05–0.07 Wm^−1^K^−1^ and excellent compressive strength of 350–570 kPa, which has great potential as an alternative insulation material for building and infrastructural applications [209]. As Sisti et al. [210] noted, the inclusion of wheat bran accelerated mycelium development, favored a more thick, hydrophobic, homogeneous surface, and could alter the mechanical behavior of the mycelium composite by serving as a reinforcing agent. This is consistent with mycelium’s capacity to effectively replace formaldehyde-based adhesives in the wood composites industry. With an optimum temperature of 160°C and a pressure of 10 mPa for 20 minutes, a bio-board constructed from *Ganoderma lucidum* waste mushroom substrates was able to achieve the highest internal bonding strength of 2.51 mPa [211]. Pre-treatment of mycelium on yellow birch veneer for eight days results in the greatest bonding performance of the interface between two wood layers, with a lap-shear strength of up to 1.74 MPa [212].

**Figure 1.**
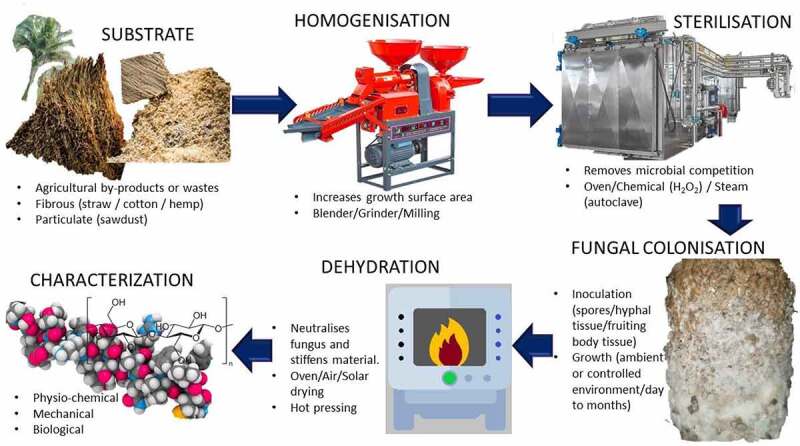
Schematic illustration of the process of mycelium-based composites.

Jones et al. [203] conducted a comprehensive evaluation of mycelium composites, synthetic foams, and wood products used in construction and discovered that mycelium composites are significantly lighter, more cost-effective, and have improved fire safety. As a result, they can be used as an alternative to conventional construction materials for thermal or acoustic cabinets, paneling, door cores, insulation, flooring, and other furnishings. Consistent with Manan et al. [197], who said that mycelium-based materials are more suited for thermal and acoustic insulation than synthetic foam and wood fibers because of their high porosity, low thermal conductivity, and low density. Figure 1 illustrates the process of mycelium-based composites.

Comprehensive usage in a building would enhance fire safety by reducing average and peak heat release, resulting in a long time to flashover with less heat and smoke [213]. Mycelium’s corresponding combustion propensity is substantially lower than that of polylactic acid (PLA) and poly (methyl methacrylate) (PMMA), showing that it is noticeably less likely to catch fire and burn fiercely [214]. *Ganoderma resinaceum* mushroom, *Miscanthus x giganteus* fibers, and potato starch were combined in a 0.3:1:0.1 ratio to produce sustainable bio-composite insulation materials with thermal conductivities ranging from 0.0882 to 0.104 Wm^−1^K^−1^, an average density of 122 kg/m^3^, and significant fire resistance under EN13501–2:2003 category EI15 [215]. Jones et al. [216] discovered that mycelium reduces oxygen levels inside the pyrolysis zone, while silica layers in the rice hull act as thermal insulation, restricting oxygen access to the unburnt material and therefore preventing volatile gas emissions into the combustion zone.

Mycelium composites are acknowledged as a viable alternative to traditional building materials, but their form-like mechanical properties, which are weaker in tensile, compressive, and flexural strength than polyurethane, wood products, and phenolic formaldehyde resin foam, as well as their highwater absorption and numerous gaps in material property documentation, make them unsuitable for structural applications [203]. Ghazvinian et al. [217] call for further investigation because the compressive strength of mycelium-based composites containing sawdust, straw, and wheat bran with the Gray Oyster mushroom (*Pleurotus ostreatus*) strain was extremely low and insufficient to replace conventional masonry materials without introducing reinforcement. However, some of them had extremely elastic behavior. Girometta et al. [218] emphasize the issue of consistency since mycelium-base composites exhibit considerable heterogeneity depending on the substrate composition, structure, fungal strain and species, and incubation conditions. Indirectly participate in further investigation of the most promising areas of research and development, notably in the field of sustainable building materials.

Currently, the primary concern is that mycelium composites cannot be used in any structural applications traditionally achieved with wood, but are better suited for applications such as door cores and certain paneling applications. In terms of fire safety, however, Mycelium composites have a significant advantage over traditional synthetic insulation materials, such as polyurethane foams and polystyrene, which are highly flammable. Mycelium composites are realistically best suited to compete with synthetic foams and wood products in thermal or acoustic insulation applications, where their low density, low cost, and fire resistance give them a significant advantage.

## 8. Green technology (fungal delignification of lignocellulosic biomass for biofuels production)

Making lignocellulosic biofuels from biomass is becoming increasingly popular. Lignocellulosic biomass is converted into fermentable sugars and subsequently biofuels by pre-treatment [219]. A precursor to enzymatic hydrolysis, delignification is required to decrease biomass levels of resistance.

Fungi agents can be classified according to the decay mechanisms they cause, such as soft rotting, white rotting, and brown rotting. Table 6 summarizes the different mechanisms of lignocellulosic biomass degradation by fungi agents.

**Table 6.** Mechanism of lignocellulosic biomass degradation by fungal agents.

Mechanism	Soft Rotting	White Rotting	Brown Rotting
Characteristic	Softened area in a wet climate, brownish area in the dry climate	Whitened, softened and moist area	Browned area, dry and brittle
Lignocellulosic biomass	Major in hardwoods, minor in softwoods	Hardwoods and softwoods	Major in softwoods, rarely in hardwoods
Degraded components	Cellulose, hemicellulose, less in lignin	Cellulose, hemicellulose, lignin	Cellulose, hemicellulose, less in lignin
Physical features	Attack of the cell wall in the proximity of hyphae	Rapid attack of cell wall entirely	Gradual attack of cell wall from the lumen, lignin in the middle lamella and secondary wall
Fungi species	*Chaetomium* *globosum, Ustulina* *deusta, Alternaria alternata*, *Thielavia terrestris*, *Paecilomyces sp., Inonotus hispidus, Rigidoporus crocatus*	*Tinea versicolor, Irpex lacteus, Phanerochaete chrysosporium, Heterobasidion annosum, Xylaria hypoxylon, Ganoderma australe*, *Phlebia tremellosa, Ceriporiopsis* *subvermispora, Pleurotus* *sp., Phellinus pini*	*Coniophora puteana*, *Gloeophyllum* *trabeum, Laetiporus* *sulphureus*, *Piptoporus betulinus*, *Postia placenta, Serpula lacrymans*
References	[220]	[221]	[222]

For biofuel generation, lignin-degrading fungi have been utilized in an alternative process rather than just thermal or chemical pre-treatments. White-rot fungi have the most effective ligninolytic mechanism. These fungi are selective for lignin breakdown over cellulose; therefore, they have high potential as pre-treatment agents for biofuel generation [223].

The oxidative enzymes such as manganese peroxidase (MnP), lignin peroxidase, and laccase are secreted by white-rot fungus, catalyzing the degradation of lignin [224]. Many white-rot fungi, such as *Dichomitus squalens, Phanerochaete chrysosporium, Phlebia radiate, Jungua separabilima*, and *Rigidoporus lignosus*, have been employed to successfully delignify wheat straw and wood [225]. Among all, *P. ostreatus* outperforms other fungi in delignifying straw [226]. When pre-treatment time was prolonged to several weeks, *P. ostreatus* degraded lignin and cellulose less selectively [227]. Hence, cellulose digestibility was found only to be stabilized at a later stage in the process. The delignification of biomass is typically fungi and feedstock specific. On the other hand, *P. cajor saju*, one of the most frequently farmed mushrooms in the world, produced the highest levels of enzyme, sugar, and ethanol during lignin degradation. These values are comparable to or higher than those described in earlier research. After 30 days of *P. sajor-caju* pre-treatment, the maximum amount of lignin degradation was 38.29%, and this was followed by a sugar yield of 71.24% and the production of 0.124 g g-1 ethanol [228]. Woody biomasses like aspen and birch benefited from longer fungal pre-treatment times, while softwood requires more pre-treatment than hardwood [229]

It might not be necessary to completely decontaminate feedstocks because white-rot fungus can exist in pollution and actively decay. The length of pre-treatment is a significant deterrent to applying fungicides. Combining fungal pre-treatment with on-farm moist storage can shorten lengthy pre-treatment timeframes. Before a physical or thermochemical pre-treatment, a fungus can be employed as a pre-treatment. The necessity for thermochemical pre-treatment can be diminished by short-term fungal pre-treatment, which can alter cell walls before visible disintegration occurs.

Brown-rot fungi pre-treatment technologies would offer tangible energy and cost benefits to the whole biofuel process. This would save energy and money throughout the whole biofuel process. Biomass is depolymerized quickly and extensively by brown-rot fungus, with minor modifications to lignin [230]. Brown rot fungal hyphae develop in the lumina of the plant cell, disrupting carbohydrate polymers away from the site of contact. Brown-rot fungi are said to release oxalic and other organic acids that lower the pH of lignocellulose, depolymerize hemicellulose and cellulose, and increase the porosity of plant cell walls.

In addition, soft-rot fungi, which include *Ascomycotina* or *Deuteromycotina*, destroy hardwood and softwood alike. However, it degrades slower than white-rot and brown-rot fungi. Soft-rot fungi destroy plant debris and wood in moist environments [231]. Soft-rot fungi can decay wood in high or low water potential environments where other fungi cannot. A wide range of wood substrates can be attacked by soft-rot fungus. These fungi are typically found in soil but can also be found elsewhere. Some soft-rot fungi create secondary wall breaches, while others cause full secondary wall erosion, leaving just the center lamella intact. Due to the difference in lignin between hardwood and softwood, soft rot is more frequent in hardwood. Soft-rot fungi produce cellulase, an enzyme that degrades cellulose in wood. Soft-rot fungi include *Chaetomium* and *Ceratocystis* species found on land, and *Lulworthia, Halosphaeria*, and *Pleospora* species found in marine and estuarine habitats.

**Figure 2.**
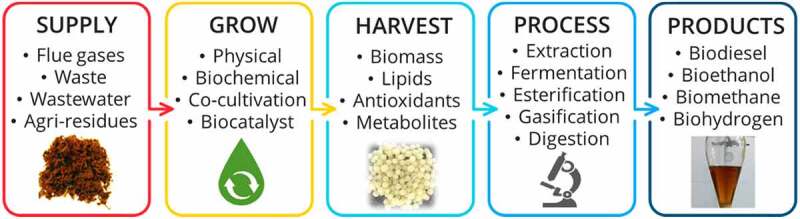
Fungal biorefinery for biofuels production.

As depicted in Figure 2, several microorganisms can break down lignocellulosic material to produce biofuels and help make fungi-based biorefineries more feasible. However, some lignocellulose polymers can be effectively broken down by lignin-digesting fungus. While soft-rot fungi concentrate on plant polysaccharides, brown-rot fungi produce enzymes that target lignin but do not degrade it. On the other hand, because they can obliterate all three parts of lignocellulose – lignin first, then cellulose – white-rot fungus has grown in favor. For the production of liquid biofuel to be cost-effective, lignin must be broken down effectively. Similar to the bio-pulping technique, pre-treatment with fungi before mild physical and chemical pre-treatment has shown synergy in improving cellulose digestibility. Overall, although less effective than thermochemical pre-treatments, pre-treatment with a fungal whole-cell biocatalyst is more energy efficient and environmentally friendly than traditional thermal or chemical pre-treatment approaches.

## 9. Wealth and economy

Pollution, malnutrition, and food insecurity are just a few of the key problems that the contemporary world has had to deal with in recent years. Fungal biomass, such as that produced by mushroom farming or artificial growing, is a potential solution to address these issues [117], as well as the cheapest protein source to compensate for malnutrition-induced protein deficit [232]. Increasing livelihood and income opportunities through regional and international trade benefits the local economy. The current section highlights an impressive explanation of the various features of fungal biomass exploitation that can preserve the planet and its inhabitants, particularly in terms of wealth and economic perspectives [233,234]. Fungal biomass has been introduced into the business for a variety of reasons, including its ease of use as an indoor crop, the ability to use vertical space to minimize land and increase waste utilization, and its status as the world’s largest protein producer per unit area and time [235,236].

Marlow Foods, Novozymes, BASF, AB Enzymes, Chr. Hansen, Bayer, Dyadic International, Roal Oy, Kerry Group, DSM, DuPont, Syngenta, and Puratos are just a few of the European companies that are capitalizing on filamentous fungi’s potential. In terms of metabolic variety, resilience, and secretory capacity, this group of microorganisms frequently outperforms bacterial and yeast-based production systems. Organic acids, proteins, enzymes, and small molecule medications, including antibiotics, statins, and steroids, have all been produced on a large scale. As a result, fungi are essential to many different sectors, such as those that produce food and feed, medicines, paper and pulp, detergents, textiles, and biofuels. However, the Aspergillus industry is promoting the myco-economic system. More citric acid is produced globally by the filamentous fungus *Aspergillus niger* than any other organic acid produced by microbial fermentation. In fact, filamentous fungi alone produce enzymes that break down plant biomass for €4.7 billion, and the market for these products is predicted to quadruple in the following ten years [7].

Table 7 lists some industries that benefit from the use of fungal biomass in the bio-economy. In comparison to other fungal-biomass-related enterprises, Novozymes has championed enzyme manufacture via *Aspergillus oryzae* [246] and achieved the largest estimated profit worth 14.95 billion USD. GNF Chemical came in second (4.7 billion USD) with *Aspergillus niger* citric acid production [4]. In the Asian region, DXN Asia (0.25 billion USD) produces *Ganoderma lucidum* functional food products, penicillin from *Penicillium chrysogenum* (North China Pharmaceutical Group Semisyntech at 0.7 billion USD), *Fusarium venenatum* [238] artificial meat by Kernel Mycofoods (0.005 billion USD) and *Mortierella alpina* food additives by Cargill Alking Bioengineering (Wuhan) at 0.007 billion USD. Meanwhile, Shenzen Hua He Sheng Technology (Water improvement by *Trametes versicolor*) and MycoCosm (Lipid manufacturing by *Umbelopsis isabellina*) are two new enterprises with innovative technologies.

**Table 7.** Some industries profiting from fungal biomass application as bioeconomy.

Prominent fungal biomass species	Industry	Company	Profit estimation (USD) Billion	Reference
*Ganoderma lucidum*	Functional food	DXN Asia	0.25	[237]
*Fusarium venenatum*	Artificial meat	Kernel Mycofoods	0.005	[238]
*Mortierella alpina*	Food additives	Cargill Alking Bioengineering (Wuhan)	0.007	[92,239]
*Aspergillus niger*	Citric acid	GNF Chemical	5	[4]
*Rhizopus microsporus*	Feed	NexPRO	0.05	[240]
*Trametes versicolor*	Wastewater and Water quality	Shenzen Hua He Sheng Technology	-	[241]
*Penicillium chrysogenum*	Penicillin	North China Pharmaceutical Group Semisyntech	0.7	[242]
*Umbelopsis isabellina*	Lipid	MycoCosm	-	[243]
*Pleurotus ostreatus*	Building Materials	MycoWorks, NEFFA, Ecovative Design, MOGU	0.01	[244,245]
*Aspergillus oryzae*	Enzyme technology	Novozymes	15	[246]

China leads the world [247] in the production of *Pleurotus eryngii, Hericium erinaceus, Flammulina velutipes, Pleurotus ostreatus, Auricularia polytricha, Auricularia auricula-judae, Wolfiporia cocos, Agaricus bisporus, Volvariella volvacea*, and *Lentinula edodes* [248], while a Philippine company Monde Nissin acquires the popular QuornTM Foods for the amount of £550 m to provide on-trend healthy fungal biomass food products. Fungal biomass produces many organic acids, life-saving medicines and antibiotics, and enzymes, and many of our foods and beverages would not exist without their fermentative abilities [7]. In 2008, the global market volume of white biotechnology goods was predicted to reach €110 billion, with that figure expected to more than quadruple by 2020 to €450 billion. Within a generation, biotechnology will create a major share of chemicals, with biotechnology goods predicted to dominate the specialty chemicals market by 2030.

In the first nine months of 2015, steroidal progestin drospirenone generated more than €0.54 billion, providing a practical illustration of the widespread production of essential building blocks for active pharmaceutical compounds in filamentous fungi. In fact, the bulk of enzymes used in the world is produced by filamentous fungi. In 2015, the market for industrial enzymes was estimated to be worth 3.5 billion euros, with Novozymes and Dupont holding 48 and 20%, respectively, of the market share [7], and other agricultural suppliers are showing interest in this area [7]. Several Aspergillus species, as well as other fungi-like *Trichoderma reesei* and *Myceliophthora thermophila* are relevant for commercial enzyme manufacturing. Because of the enormous variety of ecological niches and metabolic diversity found in mushrooms, many species, especially basidiomycetes, have substantial potential as suppliers of novel enzymes for upcoming commercial applications.

The current sustainable way of life could not be properly accomplished without the assistance of nature’s molds and mushrooms. Our future will be shaped in part by fungi, which are a part of our present. They lead the way in recycling and material transformation, and their microbial counterparts cannot match their biosynthetic skills. Fundamental and applied fungi science give us new ideas for ensuring future human, animal, and plant health as well as solutions for moving from a petroleum-based economy to a bio-based circular economy. They also open up new opportunities for food security as demand rises due to an expanding human population. As described in this review, fungi science has already helped to achieve 11 out of the 17 UN development objectives, and its significance will only increase in the future. Industries, agriculture, and the environment will gain the most from fungal biomass biotechnology applications [249]. In the production of important bioactive chemicals, including terpenes, flavonoids, and essential oils, as well as for bioremediation and the treatment of pollutants, fungi play a crucial role. They play a crucial role in the synthesis of alkaloids, azadirachtins, and cytochalasins.

The use of agrochemicals indiscriminately, excessive bush burning, soil tilling, and excessive deforestation all contribute to environmental degradation in agriculture. Simply said, sustainable agriculture attempts to use soil-friendly practices and technology with less usage of nonrenewable resources. The systematic and effective use of biological materials, such as fungi, is required by appropriate agriculture to increase the mobilization of critical nutrients, especially in the management of disease and pests, while increasing overall plant yield. This is necessary to meet the global needs and demand for food sufficiency. Fungi, in the meantime, are crucial to the environment’s ability to break down dead and decayed materials. This characteristic of fungi allows humans to use them as a bioinoculant for the breakdown of pollutants.

## 10. Challenges and limitation

**Figure 3.**
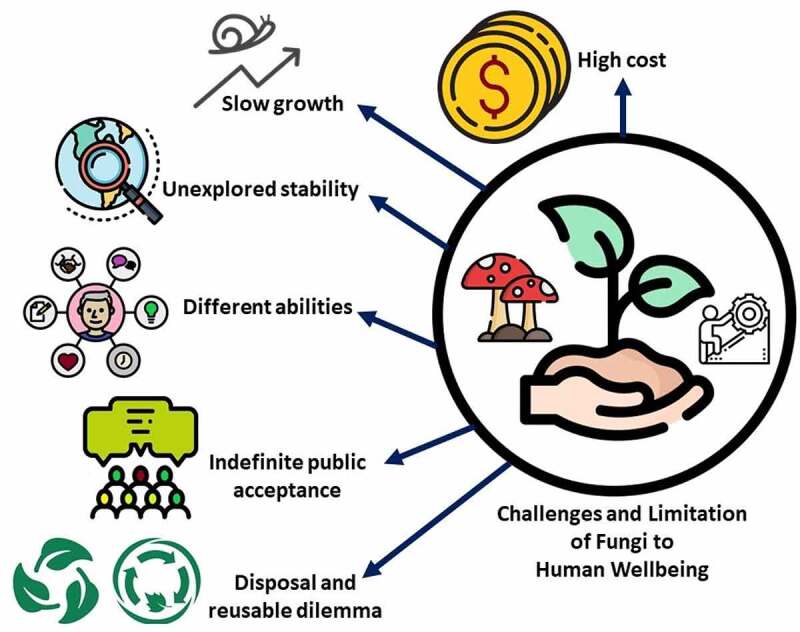
Challenges and limitation of fungi to human wellbeing.

Despite the numerous advantages of fungi in contributing to human wellbeing, it poses some disadvantages, like other applications in the world (Figure 3). Having been said, we believe that the positives outweigh the setbacks, whereby it can be asserted with confidence that the negative sides are nothing alarming, dangerous, or hazardous. We identify the key aspects of the challenges and limitations of fungal adaptation on a wide scale.

- Cost of fungal process

A high-scale liquid fermentation system must be used to produce fungal biomass in bulk, safely, affordably, and with a consistent quality for mass consumption. The sophisticated bioreactor systems employed by food corporations like Yakult and Nestle require fully qualified personnel in the fields of industrial biotechnology, microbiology, and fermentation technology.

- Growth of fungi

Use of fungus biomass, such as fruiting bodies or mycelium pellets, can take a long time to generate in large quantities and demands a lot of resources and upkeep. The fungi had to be in ideal environments, such as temperature, pH, and availability of carbon sources, or else their performance would suffer. The growth rate of fungus may be inhibited by contaminants in high-contaminated home water treatment; even if remediation occurs, the fungus’s long-term viability will be reduced.

- Stability of fungi

As fungi have the potential for a wide application, the resiliency of fungi to harsh conditions is yet to be explored. The decomposition of fungal biomass and the destruction of functional enzymes are highly likely when the fungi are introduced to (or in) a non-ideal fungal environment.

- Different fungal species have different abilities.

With thousands of fungi species available, the ability of each varies for each application. Which strain or species of brown rot, white rot, or soft rot is good for the intended purposes and objectives? For example, bioremediation cannot be neglected in the treatment of water by white-rot fungal species alone; even studies reveal a remarkable succession of removal since each fungi species has a varied potential for producing bio-adsorbent. Other elements, however, may have an impact on the efficiency of fungal bioremediation.

- Public acceptance of fungi

The efficacy of fungus in the treatment of residential water is undeniable. The public, on the other hand, may be skeptical of the technology because it is a novel treatment. Another issue that could develop is the possibility of biomass residue infiltrating the water during the treatment. The water’s safety can be questioned because there aren’t many studies confirming that treated home water is safe to drink. Even so, it’s possible that it’s only safe for outward daily use and necessities.

- The fate of the fungi in terms of disposal and reusable

Fungi are one of the biodegradable substrates; thus, they won’t last forever in the water. However, it may become a problem if a large number of them die, disrupting the environment within the water. Instead of treating the water to make it cleaner, other issues may occur. Considering the long-term viability of fungal biomass in water treatment, the immobilization of fungi may be a viable solution. The immobilization can be done on various inorganic substances that the fungus can solidly cling to. Furthermore, inorganic compounds can last longer than organic chemicals throughout treatment.

## 11. Conclusion

Fungal biomass is paving its way to the future. The ability to produce in bulk, safe, cheap, environmentally friendly, and most importantly, found to be consistently produced at high quality are the obvious advantages of fungi, imposing themselves as attractive alternatives to bacteria and algae. We are adamant that fungi support and are consistent with the UN’s Sustainable Development Goals. Goal #3, ‘Good health and wellbeing,’ Goal #6, ‘Clean water and sanitation,’ and Goal #7, ‘Affordable and clean energy,’ all have direct effects. Utilizing fungi is consistent with the shorter-term goals of the 2030 Agenda for Sustainable Development, including Goal 2.3, which calls for doubling agricultural productivity, and Goal 2.4, which calls for ensuring sustainable food production systems and putting in place resilient agricultural practices that boost productivity and production. The adaptation of fungi as tertiary/polishing water and wastewater treatment is definitely hit by Goal 6.3 to improve water quality by reducing pollution, minimizing the release of hazardous chemicals and materials, halving the proportion of untreated wastewater, and substantially increasing recycling and safe reuse globally. Goal 7b, which calls for upgrading technology and expanding infrastructure to provide modern, sustainable energy services to all developing nations, may materialize and be accomplished by 2030 with the development of biofuel production using fungi. Goals 9.5 and 9.b, which focus on improving scientific research, modernizing technological capabilities, and supporting domestic technology development, research, and innovation, respectively, are in line with the requirement to support technological breakthroughs from laboratory experiments to large-scale applications. Numerous fungus species and strains have been successfully applied, but the majority have not yet been applied to industrial processes, according to reports from around the world. The potential for the commercialization of fungi is enormous, either in terms of aiding or playing a crucial part in the production process or in terms of directly being used in its processed forms. With the sole purpose of promoting the benefits and welfare of both humans and the environment, the flourishing development of fungal-based medicines offered and stimulated opportunities to be developed in a wider scope of application.

Despite the overwhelming advantages of fungal applications, adaptation to big-scale industrial applications has proved challenging. The paradigm shift into new technologies is crucial, with the key players yet to fully grasp the benefits of fungal-based methods, hindering the full speed of direct application. Translating language between technical science-based terms and business expressions is one of the challenges to smoothly transitioning from laboratory to market. A bridging platform with a good understanding of technical and business terms is seen as essential in bringing fungi as biomaterials of the future.

Although reports have shown that fungal-based may improve the performance of one activity, the substantial initial capital cost needed for refurbishment, restricted allowable space and time for technology transition without financial losses, the reluctance to disrupt existing practices or processes, and the lack of support in terms of national policies and plans, are some of the key issues that need to be addressed before wider commercialization of fungi in the industry.

## 12. Methodology

The study does not require ethical approval because the manuscript is in the form of a review article.
